# Variant load of mitochondrial DNA in single human mesenchymal stem cells

**DOI:** 10.1038/s41598-024-71822-4

**Published:** 2024-09-09

**Authors:** Daniel Hipps, Angela Pyle, Anna L. R. Porter, Philip F. Dobson, Helen Tuppen, Conor Lawless, Oliver M. Russell, Doug M. Turnbull, David J. Deehan, Gavin Hudson

**Affiliations:** 1 The Newcastle Upon Tyne Hospitals NHS Foundation, Newcastle upon Tyne, UK; 2 Wellcome Centre for Mitochondrial Research, Translational and Clinical Research Institute, Newcastle University, Newcastle upon Tyne, UK; 3 Wellcome Centre for Mitochondrial Research, Biosciences Institute, Newcastle University, Newcastle upon Tyne, UK

**Keywords:** Mesenchymal stem cells, Somatic mtDNA variation, Osteoporosis, Bone disease, Genomic analysis, PCR-based techniques, Next-generation sequencing, Mesenchymal stem cells, Bone, Osteoporosis

## Abstract

Heteroplasmic mitochondrial DNA (mtDNA) variants accumulate as humans age, particularly in the stem-cell compartments, and are an important contributor to age-related disease. Mitochondrial dysfunction has been observed in osteoporosis and somatic mtDNA pathogenic variants have been observed in animal models of osteoporosis. However, this has never been assessed in the relevant human tissue. Mesenchymal stem cells (MSCs) are the progenitors to many cells of the musculoskeletal system and are critical to skeletal tissues and bone vitality. Investigating mtDNA in MSCs could provide novel insights into the role of mitochondrial dysfunction in osteoporosis. To determine if this is possible, we investigated the landscape of somatic mtDNA variation in MSCs through a combination of fluorescence-activated cell sorting and single-cell next-generation sequencing. Our data show that somatic heteroplasmic variants are present in individual patient-derived MSCs, can reach high heteroplasmic fractions and have the potential to be pathogenic. The identification of somatic heteroplasmic variants in MSCs of patients highlights the potential for mitochondrial dysfunction to contribute to the pathogenesis of osteoporosis.

## Introduction

Mesenchymal stem cells (MSCs) are the non-haematopoietic progenitor cells of the musculoskeletal system^1^. MSCs have the capacity to differentiate into mesodermal lineages such as osteocytes, adipocytes, and chondrocytes as well ectodermal and endodermal lineages and, are thus critical to the generation, maintenance and repair of skeletal tissues and bone^1^. However, the regenerative potential of MSCs decreases during ageing^2,3^. Thus, therapeutic strategies that either remove, rejuvenate, or replace senescent MSCs have been proposed to repair bone and cartilage injuries^4^ or to mitigate the degeneration that occurs during age-related diseases such as osteoarthritis and osteoporosis^5,6^. However, significant challenges remain. MSCs are relatively rare cells and are difficult to isolate and culture^7^. In addition, there is uncertainty regarding successful delivery to affected tissues^8^ and MSCs are often rapidly removed after transplantation which limits their therapeutic ability^9,10^.

Primary osteoporosis occurs when the destruction of bone by osteoclasts overtakes the formation of bone by osteoblasts^11^ and is associated with the senescence of MSCs^12^. The reduction in bone mass and microarchitecture deterioration results in a loss of strength leading to an increased risk of fragility fractures with advancing age^13–15^, with bone mass and mineral density peaking in the third decade before declining with advancing age^16^.

Decreased mitochondrial function is a hallmark of human ageing^17^ often accompanied by an increase in somatic mitochondrial DNA (mtDNA) variation^18^, and is a component of several age-related bone diseases including osteoporosis and osteoarthritis^19,20^. Animal models of increased mtDNA mutagenesis show evidence of osteoporosis^21^ accelerated bone loss and bone formation, with a corresponding reduction in osteoblasts and increased osteoclasts^20^. This age-related bone loss correlates with increased mitochondrial dysfunction in MSCs^20^. In humans, osteoblast populations and mitochondrial deficiency both increase with age^16^, and it has been suggested that mitochondria regulate the balance between osteoclast/osteoblast activity^19^. These observations are consistent with the animal models and suggest that the accumulation of somatic mtDNA variants may play a significant role in the pathoetiology of osteoporosis in humans.

However, investigating whether somatic mtDNA variants have a role in age-related bone disease is challenging. MtDNA mutations are known to accumulate in stem cell populations^22^, however, MSCs are relatively sparse and there are challenges relating to accurate and efficient isolation^22^ Additionally, mtDNA is polyploid^23^ and variants can be present on all mtDNAs (a state known as homoplasmy) or only in a fraction of mtDNAs (known as heteroplasmy)^24^. To manifest a phenotype, a heteroplasmic variant must reach a specific threshold, which can range between 60 and 90%^25^. In addition, somatic mtDNA variants are acquired, often initially in stem cell compartments and then persisting in differentiated cells^26^, and can increase in heteroplasmic fraction during ageing due to clonal expansion^27^. Thus, to be confident of the understanding of mtDNA variants in age-related diseases, we must investigate individual cells.

Understanding the mutational landscape of MSCs could explain the increase in respiratory deficient osteoblasts seen in previous studies and thus play a role in the pathogenesis of osteoporosis. Here we present a proof-of-concept study, using single-cell mtDNA sequencing to investigate the landscape of somatic mtDNA variation in MSCs, the precursors to human osteoblasts.

## Materials and methods

### Cohort

Bone marrow samples were taken at the time of elective hip arthroplasty and other routine orthopaedic surgeries from 13 individuals (5 males, mean age = 51.0 ± 26.4, and 8 Females, mean age = 78.8 ± 15.9) undergoing elective orthopaedic surgery for either trauma or osteoarthritis between 2017 and 2019 from the Newcastle upon Tyne Hospitals (STable 1). Ethical approval for the use of adult human samples was gained as an adjunct to the Newcastle Bone and Joint Biobank (REC reference 14/NE/1212, IRAS project ID166522 and Newcastle University reference 8741/2016). All patients gave informed consent to the use of tissue as part of the ethical approval requirements and experiments were performed in accordance with relevant guidelines and regulations.

## MSC isolation and lysis

Bone marrow samples were split into two equal-volume aliquots, one for single cell isolation and a second to generate a ‘consensus sample’ for comparison. For one aliquot, mononuclear cells were concentrated and separated from the rest of the marrow (X ml) using a density gradient (a 1.077 g/ml Lymphoprep™ density gradient medium, StemCell Technologies)^28^. This removed contaminants from the sample such as red cells, fat and multinucleated cells which were not of interest. MSCs were isolated within the Lymphoprep™—marrow interphase. MSCs were concentrated within the mononuclear cells to facilitate Fluorescence-activated cell sorting (FACS, SFigure 1). Individual MSCs were sorted from the mononuclear cell pool by FACS using lineage-specific cell markers. Purified MSCs were defined as positive for cell surface markers, CD73, CD90, and CD105 while being negative for CD34 and CD45 (based on the MSC Phenotyping Cocktail Kit, anti-human, REAfinity™ and previous work^29–31^. Individual MSCs were FACS sorted into wells of a 96-well microtiter plate using electrostatic deflection (taking ~ 20 min per 96 cells). Each MSC was lysed in 15 µl single-cell lysis buffer, containing 0.5 M Tris–HCl, 0.5% Tween 20, 1% Proteinase K, pH 8.5. Lyses were centrifuged at 150G for 2 min at room temperature to ensure the cell was submerged. MSCs were lysed for 3 h at 55 °C.

In total, 146 MSCs were extracted across the 13 patient bone marrow samples (> 10 cells per sample, STable 2). The remaining aliquot (estimated as ~ 1 million cells) was also lysed using the same conditions and used as a per sample reference for mtDNA sequencing.

### Mitochondrial DNA amplification and deep sequencing

As in previous work^32^, and to ensure efficient and accurate amplification from low-concentration single-cell extracted DNA (i.e., fg/ml), mtDNA was enriched using five overlapping long-range PCR amplicons (rCRS, or GenBank ID NC_012920.1, positions as—Set 1: m.323-343 and m.3574-3556; Set 2: m.3017-3036 and m.6944-6924; Set 3: m.6358-6377 and m.10147-10128; Set 4: m.9607-9627 and m.13859-13839 and Set 5 m.13365-13383 and m.771-752). As in previous work^32^, primer efficiency and specificity were assessed as successful after zero amplification of DNA from Rho0 cell lines, to avoid the unintended amplification of nuclear pseudogenes (nuclear-mitochondrial segments or NUMTs^33^). Rho0 cell lines were provided by the Mitochondrial Research Group, Institute of Genetic Medicine, Newcastle University.

Amplified products were assessed by gel electrophoresis, against DNA +ve and DNA −ve controls, and quantified using a Qubit 2.0 fluorimeter (Life Technologies, Paisley, UK), purified using Agencourt AMPure XP beads (Beckman-Coulter, USA) and pooled in equimolar concentrations and re-quantified. Pooled amplicons were tagmented, amplified, cleaned, normalized, and pooled into a single Illumina library using the Nextera XT DNA sample preparation kit (Illumina, CA, USA). Multiplex pools were sequenced using MiSeq Reagent Kit v3.0 (Illumina, CA, USA) in paired-end, 250 bp reads. Post-sequencing data, limited to reads with QV ≥ 30, were exported for analysis. In total, 140 cells were successfully amplified, sequenced, and moved forward for bioinformatic analysis (STable 2).

### Bioinformatic analysis

Post-sequencing FASTQ files from 140 samples were analysed using an in-house bioinformatic pipeline^32,34,35^. Briefly, sequence reads were quality checked using FastQC (v. 011.8), mapped against genome version GRCh38 (including NC_012920.1), using BWA (invoking –mem, v. 0.7.17)^36^ sorted and indexed using Samtools (v.1.12)^37^. All duplicated reads from the resulting bam files were marked using Picard (v.2.2.4). mtDNA variants (mtSNVs) were called using bcftools (v1.10.2) and Mutserve (v.2.0.0) accordingly^38,39^. mtSNVs with base quality (--baseQ) over 30 and minimum heteroplasmy level (--level) 0.02 were called. Low-quality variants, present in low-complexity regions^40^ were not included in comparative analysis (e.g., 66–71 bp, 300–316 bp, 513–525 bp, 3106–3107 bp, 12,418–21,425 bp, 16,181–16,194 bp). The remaining variants were annotated via ENSEMBL VEP v107^41^. Heteroplasmic fraction (HF) is defined as the proportion of mtDNA variant allele depth relative to reference allele depth. Homoplasmic variation was defined as HF > 0.98 (98%). Heteroplasmic variation was defined as HF > 0.02/< 0.98 (2% and < 98%). Heteroplasmic counts are based on the number of heteroplasmies per cell. To ensure high-quality comparisons, we invoked strict post-bioinformatic quality control (QC). We removed samples if mtDNA (rCRS or GenBank ID NC_012920.1) coverage was < 99% at a minimal depth of 1500×.

After filtering for low coverage, 99 cells remained (ensuring at least > 3 cells per sample) and were taken forward for analyses (STable 3). After variant calling, each single cell was compared to the consensus sample to confirm homology through homoplasmic variant sharing (e.g., mtDNA haplogroups^42^, STable 4) and to assess potential contamination. Haplogroups were determined using Haplogrep 3 (v3.2.1, confidence > 0.9^43^, and all samples were of European ancestry (STable 4).

### Statistical analysis

Somatic variants were defined as heteroplasmies (> 2% and < 98%) present in 1 cell and absent in the consensus (STable 4) and each of the other cells. All analyses were carried out in R (v3.4) using data-appropriate tests (detailed in text). Statistical significance was set at < 0.05. Multiple significance correction can be too conservative for a discovery study, particularly when testing a priori hypotheses with variables that are not all independent^44^. Thus, unless specified in the text, we report unadjusted P values for reasons well documented in the literature^44^.

## Results

### MSC identification and isolation

Through a combination of density gradient and flow cytometry cell sorting (FACS), we were able to successfully isolate 146 single-MSCs across the 13 patient bone marrow samples (STable 2). All single-MSCs (100%) and the 13 consensus samples were accurately enriched for mtDNA and prepared for next-generation sequencing (NGS). After NGS, we obtained high-quality sequence data (covering > 99% mtDNA at > 1500 at > 1500×) for 99 MSCs and each consensus sequence (STable 2), with 47 MSCs removed due to low coverage (STables 2 and 3).

### mtDNA variation

Prior to characterising the single MSCs, we analysed the consensus sequence data. Twelve of the 13 patient samples harboured heteroplasmic variation (ranging between 1 and 7 heteroplasmic variants with between ~ 0.02–0.94 heteroplasmy fractions, HF, STable 4). None were previously reported as deleterious (Mitomap.org). Consensus sample homoplasmic variation was used to confirm ancestry and single-MSCs/consensus similarity (all > 99% similarity).

**Fig. 1:**
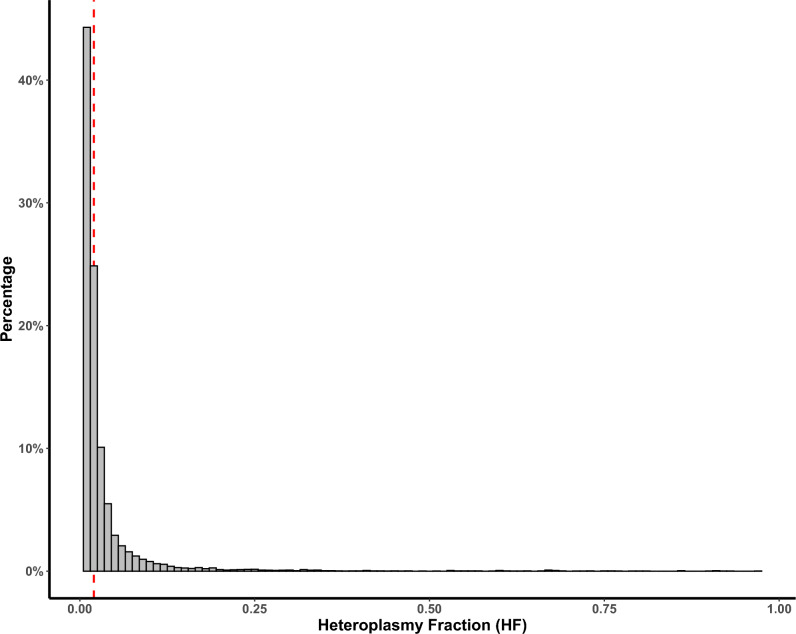
Histogram of somatic heteroplasmic variation in 13 patient samples. Frequency histogram of heteroplasmy fraction (HF) of all somatic heteroplasmic variants (HF > 0.00/< 1.00, n = 7849 variants) identified in 99 cells from 13 samples. The red line indicates a HF of 0.02. The vast majority of somatic heteroplasmic variation (n = 5,372 or 68.4%) had a HF < 0.02. 2,477 variants (31.6%) were taken forward for further analysis (SDataset 1).

Next, we investigated somatic heteroplasmic variation in each of the isolated single-MSCs (n = 99), using each corresponding consensus sequence data as a sample-specific reference. In total, we identified 7,849 somatic mtDNA variants with a HF > 0.00/< 0.98. The majority of these variants had a HF < 0.02 (5,372 or 68.4% of total somatic variants, Fig. 1). For further analysis, and similar to previous work^32,45,46^, we set a conservative HF threshold of 0.02, selecting 2,477 variants (or 31.6%) for further analysis (Fig. 1 and SDataset 1).

**Fig. 2:**
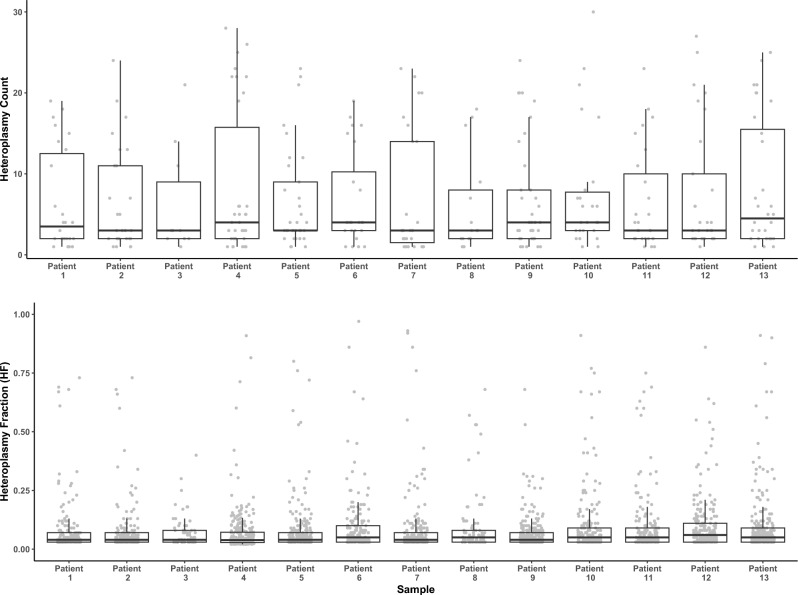
Somatic heteroplasmic count and heteroplasmic fraction distributions are similar across all 13 patient samples. Boxplots and strip charts showing the count of somatic heteroplasmies per cell per sample (*upper*) and the heteroplasmic fraction (*lower*, HF > 0.02/< 0.98) of each somatic heteroplasmic variant detected in each single-MSC grouped by sample. We observed no significant difference in heteroplasmy counts or heteroplasmy fraction distribution between samples (for each, ANOVA *p* > 0.05 and Dunnett’s *p* > 0.05 with patient 1 as reference). Boxes denoted 25th and 75th percentile, whiskers indicate 95^th^ percentile and the horizontal line indicates the median.

Although there was some variability between single-MSCs within the same bone marrow sample, likely a result of cell-specific clonal expansion^18,26,47^, the distribution of heteroplasmic variant counts were highly similar between patient samples (Dunnett’s *p* > 0.05, Fig. 2 and STable 5). Additionally, although some specific cells harboured comparatively high-fraction variants (61 variants with HF > 0.5 across 12 out of 13 samples), the overall distribution of heteroplasmic fractions within single-MSCs was highly similar between patients (ANOVA *p* = 0.07 and Dunnett’s Test with Patient 1 as the reference category *p* > 0.05 for all groups) and was typically below 10% (Fig. 2 and SDataset 1). Further analysis of somatic heteroplasmic variants showed that 33.95% were T > C substitutions, 29.75% were A > G, 20.67% were C > T and 9.12% were G > A. The remaining possible polymorphisms (A > C, A > T, C > A, C > G, G > T, G > C, T > A and T > G) represented 6.5% of the variants detected (STable 6).

**Fig. 3:**
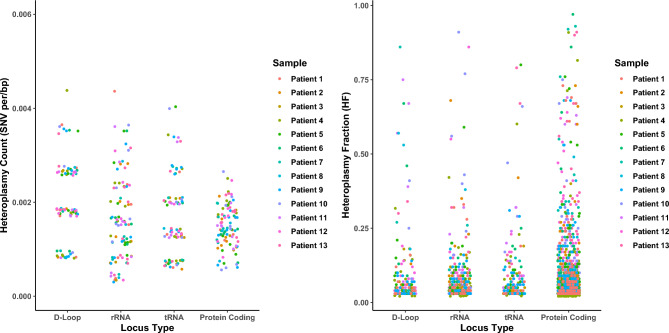
Somatic heteroplasmic count and heteroplasmic fractions stratified by locus. Strip charts showing count of heteroplasmies (HF > 0.02/< 0.98) per cell per sample after normalisation by locus length (total region bp lengths: D-loop = 1124, rRNA = 2511, tRNA = 1486 and Protein Coding = 11,382) (*left*) and the heteroplasmic fraction (HF > 0.02/< 0.98) of somatic heteroplasmies (*right*) grouped by locus type. As expected, there is a higher frequency of heteroplasmic variants in the D-Loop compared to rRNA, tRNA and protein-coding regions (ANOVA p = 8.2 × 10^–5^, Dunnett’s p, D-Loop v rRNA = 0.002, D-Loop v tRNA = 0.011, and D-Loop v protein coding = 0.001). There was no significant difference in overall HF between loci (ANOVA *p* = 0.115), However, rRNA HFs appear higher when compared to D-Loop as a reference (Dunnett’s p, D-Loop v rRNA = 0.038). tRNA and protein HFs distributions were not significantly different (Dunnett’s p, D-Loop v tRNA = 0.203 and D-Loop v protein coding = 0.082).

Previous work has suggested that somatic mtDNA variation often occurs in regional hotspots^32,48–51^, particularly within the mtDNA control region (m.16024-576^52^). Thus, we next investigated whether single-MSCs showed a similar trend. Similar to others^49,51^, we observed a significant overrepresentation of somatic heteroplasmic variants in the D-Loop (m.16024-576) when compared to other loci (Fig. 3, Dunnets p, all comparisons < 0.05). However, we found no significant difference in overall HF between loci, (Fig. 3, ANOVA *p* = 0.115). Although, rRNA HFs were significantly higher when compared to D-Loop as a reference category (Dunnett’s p, D-Loop v rRNA = 0.038), tRNA and protein-coding variant HF distributions were not significantly different (Dunnett’s p, D-Loop v tRNA = 0.203 and D-Loop v protein coding = 0.082). This is in line with previous work^53^ and suggests that heteroplasmies in the rRNA genes (MT-RNR1 and MT-RNR2) are more tolerated as they must reach a higher HF to exert a biochemical defect^54^. Overall, these data suggest that although there is a regional accumulation of mtDNA variation, selection appears uniform across loci.

**Fig. 4:**
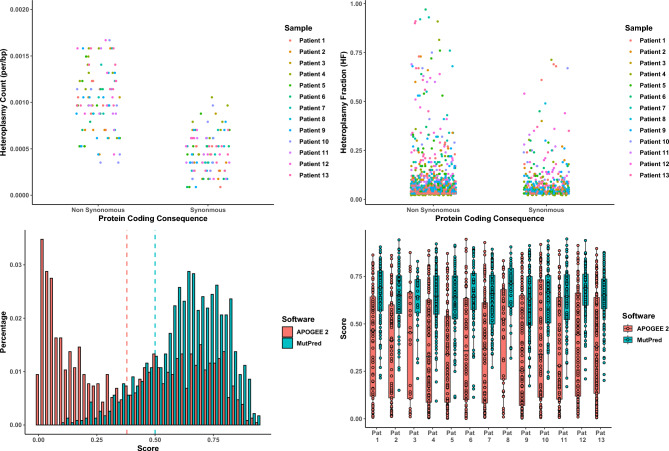
Non Synonomous and synonomous somatic heteroplasmic count and heteroplasmic fractions stratified by locus. *Upper Left:* Strip chart showing the heteroplasmy (HF > 0.02/< 0.98) counts per cell per sample after normalisation by locus length (total region bp lengths: D-loop = 1124, rRNA = 2511, tRNA = 1486 and Protein Coding = 11,382). There was a significant increase in non-synonymous variant counts across all 13 individuals (Mann Whitney U, *p* < 0.001). *Upper Right:* Strip chart showing the distribution of protein-coding variant heteroplasmy fractions (HF > 0.02/< 0.98) after stratification into non-synonymous and synonymous variants. We observed no significant difference in HF between non-synonymous and synonymous variants (Mann Whitney U, *p* > 0.05). *Lower left*: Histogram of MutPred^57^ and APOGEE 2^58^ scores showing a significant skew towards pathogenicity (MutPred score > 0.50^57^, Kurtosis = 3.17, Shapiro-Wilks Normality Test *p* < 0.001 and APOGEE 2 score > 0.38^58^, Kurtosis = 1.57, Shapiro-Wilks Normality Test *p* < 0.001). Dotted red lines indicate MutPred and APOGEE thresholds (0.5 and 0.38 respectively). *Lower right*: Boxplots of MSC somatic heteroplasmic MutPred and APOGEE 2 scores indicating that the majority of samples harbour cells with potentially pathogenic variants (MutPred^57^ pathogenic = 949 variants or 82% and APOGEE 2^58^ = pathogenic 593 or 51%).

Finally, to explore the potential functional consequence of the identified somatic heteroplasmic variants in MSCs, we stratified protein-coding variants (Fig. 3) into variants predicted as non-synonomous and synonymous (Fig. 4). Although we found no significant difference in the HFs achieved by non-synonymous versus synonymous variants (Mann Whitney U, *p* > 0.05, Fig. 4), we did identify a significant increase in non-synonymous variant count (Mann Whitney U, *p* < 0.001, Fig. 4). Similar to previous work^55,56^, we calculated a MutPred score^57^ and APOGEE 2^58^ scores of each of the coding region somatic heteroplasmic variants. APOGEE 2 appeared more conservative (Fig. 4), however, both tools showed a significant skew towards pathogenicity (MutPred score > 0.50^57^, Kurtosis = 3.17, Shapiro-Wilks Normality Test *p* < 0.001 and APOGEE 2 score > 0.38^58^, Kurtosis = 1.57, Shapiro-Wilks Normality Test *p* < 0.001) with the majority of somatic variants identified in MSCs from each sample predicted to be pathogenic (MutPred^57^ pathogenic = 949 variants or 82% and APOGEE 2^58^ = pathogenic 593 or 51%, Fig. 4). Taken together, these data suggest that, similar to previous work^59,60^, somatic non-synonymous variation is relatively frequent in single MSCs from aged individuals and has the potential to be pathogenic, accepting that HFs are similar between non-synonymous and synonymous variants^51^.

## Discussion

Here we present a proof-of-principle study of the landscape of somatic mtDNA heteroplasmic variation in MSCs isolated from patient bone marrow (BM). Somatic heteroplasmic variation appears pervasive in MSCs, with multiple variants present in individual cells, often at high HF and with the potential to be deleterious. Understanding the mtDNA variant landscape of MSCs, the progenitors of cells of the musculoskeletal system^1^, has the potential to inform our understanding of bone-related conditions that exhibit age-related mitochondrial dysfunction.

Despite their critical role in the generation, maintenance and repair of skeletal tissues and bone, MSCs are relatively sparse, accounting for ~ 0.01% of the cellular population of bone marrow^61^. Thus, the accurate isolation of in-tact, uncontaminated, MSCs from BM is challenging^62^. Although MSCs can be isolated and cultured^62^ which would facilitate investigations of MSC mtDNA variation, previous work has shown that heteroplasmic fractions (HF) can dramatically change due to genetic drift and clonal expansion during culture^23,63,64^.

To overcome these challenges, we devised a FACs-based strategy that utilised both positive and negative selecting cell surface makers to isolate individual and intact BM-derived MSCs from all patient samples (n = 146 across 13 patient samples) for downstream next-generation-sequencing (NGS) of mtDNA. The success of single-cell sequencing and the accuracy of heteroplasmy detection is dependent on sample, template quantity and quality^33^. BM-derived MSCs are estimated to have ~ 800 copies of mtDNA^65^, which is on par with or higher than previous single-cell mtDNA sequencing experiments^66–68^. In line with this, we were able to successfully isolate and enrich mtDNA from all isolated MSCs. However, despite successful amplification of mtDNA in all cells, 47 MSCs fell below the quality control threshold, typically due to significant portions of low mtDNA coverage. In this instance, we preferred to offset the reduction in sample number, rather than reduce the confidence in heteroplasmy detection. For cells passing QC, we were able to identify somatic heteroplasmic variants. The count per cell and heteroplasmic fractions (HF) were comparable between samples. Overall, and similar to others^69^, the highest number of variants (after normalisation for sequence length) were observed in the D-Loop, followed by rRNA, protein-coding and finally tRNA regions. MSCs did appear to harbour an overrepresentation of non-synonymous somatic heteroplasmies and whilst the HFs were typically low (< 0.25), the vast majority were predicted to be pathogenic and thus have the potential to be functionally relevant.

This work provides insight into the origin of mtDNA variants in blood, bone, and skeletal tissues. MSCs respond to multiple different stimuli and differentiate into multiple cell lineages, such as bone, cartilage, adipose, muscle, tendon and stroma, or self-renew^70^. Tracking clonal expansion from one cell to daughter cells from in vivo samples is therefore very challenging. Thus, we do not know whether variants detected in MSCs play a role in respiratory deficiency seen in human osteoblasts, but it would be logical to conclude this could be the case. Taken together, our data suggest that MSCs can be accurately isolated and used to study mtDNA. Additionally, our data suggests that potentially pathogenic somatic heteroplasmic variation is relatively common in MSCs, albeit it at relatively low levels.

This is of particular interest given observations of respiratory chain deficiency in human osteoblasts during advancing age^71^. This mirrors deficiencies seen in in other disorders that are attributed to an age-related accumulation of somatic mtDNA variants^22,23,72^ and whilst investigations in dental tissue-derived MSCs identified some specific mtDNA pathogenic variants in MSCs^65^, work in BM-derived MSCs has been limited, restricted by sample availability. Our work shows that somatic variation does occur in early adulthood (i.e. patients 1 and 2 were aged 22 and 25 respectively at sampling) and similar to others^31^, suggests that studying clonal expansion of mtDNA variation in bone diseases could unlock some of the hidden pathology of these diseases. It is worth noting that investigating the dynamic relationship between somatic heteroplasmic variation and mitochondrial function is in itself challenging^73^ and would be compounded by the variation often observed between samples across age ranges^74^ and would require large sample numbers. For example, > 200 colon samples were required to establish the relevance of somatic heteroplasmic variants to the respiratory chain deficiency observed during human ageing^74^.

## Conclusions

Several lines of evidence^18,21,75,76^ suggest that an age-related accumulation of somatic mtDNA variants contributes to disease^20,34,71,74^, including bone disease. In this proof-of-concept study, we provide evidence that somatic heteroplasmic variants are common in patient-derived MSCs, can reach high heteroplasmic fractions and have the potential to be pathogenic, overcoming the significant challenges of isolating and investigating patient-derived MSCs. This work suggests that a larger, cross-sectional study of mtDNA variation in bone disease is warranted.

## Supplementary Information

Supplementary Information 1.

Supplementary Information 2.

## Data Availability

Data Availability The datasets generated and/or analysed during the current study are available in the European Nucleotide Repository (Accession: PRJEB79104).
