# Supplementary material for: Variant load of mitochondrial DNA in single human mesenchymal stem cells
Source: Sci Rep. 2024 Sep 9;14:20989. doi: 10.1038/s41598-024-71822-4 (PMC11385243; doi:10.1038/s41598-024-71822-4)
Supplement: Supplementary file 2 — Supplementary Information 2. [file 41598_2024_71822_MOESM2_ESM.docx]

## ***Supplementary Information***

**Contents:**

Supplementary Figure 1

Supplementary Table 1

Supplementary Table 2

Supplementary Table 3

Supplementary Table 4

Supplementary Table 5

Supplementary Table 6

Supplementary Figure 1

**Supplementary Figure 1.**

**Supplementary Figure 1 | Flow cytometry gating strategy used to sort single MSCs from bone marrow.**

The MSC population is circled in red. The MSC population is first defined the side scatter (SSC-A) representing granularity and forward scatter (FSA-A) representing size. Further delineation is then made using positive expression of CD73, CD90, and CD105, and the negative lineage markers CD34, CD45, and CD14.Flow cytometry gating strategy to sort single MSCs from fresh bone marrow. The MSC population is circled in red. The MSC population is first defined the side scatter (SSC-A) representing granularity and forward scatter (FSA-A) representing size. Further delineation is then made using positive expression of CD73, CD90, and CD105, and the negative lineage markers CD34, CD45, and CD14.

**Supplementary Table 1.**

*Summary of 13 patients undergoing elective surgery where a bone marrow sample was taken. The table shows age at surgery (years), sex (M=male, F=female), date of surgery, reason for surgery and co-morbidities.*

| **Sample** | **Age** | **Sex** | **Surgery** | **Date of Surgery** | **Reason for Surgegry** | **Co-morbidities** |
| --- | --- | --- | --- | --- | --- | --- |
| Patient 1 | 22 | M | Intramedullary nail and bone graft | 27/03/2019 | Leg length discrepancy  after trauma | None Reported |
| Patient 2 | 25 | M | Total hip replacement | 09/03/2017 | Post traumatic acetabular fracture non-union | None Reported |
| Patient 3 | 45 | F | Total hip replacement | 02/04/2019 | Osteoarthritis | None Reported |
| Patient 4 | 53 | F | Total hip replacement | 16/01/2019 | Osteoarthritis | Hypertension |
| Patient 5 | 61 | F | Total hip replacement | 24/11/2017 | Osteoarthritis | Hypertension |
| Patient 6 | 62 | M | Total hip replacement | 02/05/2019 | Osteoarthritis | Type II diabetes mellitus, Hypertension |
| Patient 7 | 64 | M | Total hip replacement | 11/04/2019 | Osteoarthritis | Chronic kidney disease, Type II diabetes mellitus |
| Patient 8 | 74 | F | Total hip replacement | 11/04/2019 | Osteoarthritis | Atrial fibrillation, pulmonary hypertension, iron-deficiency anaemia |
| Patient 9 | 78 | F | Total hip replacement | 24/11/2017 | Osteoarthritis | Hypertension |
| Patient 10 | 82 | M | Total hip replacement | 03/04/2019 | Osteoarthritis | chronic obstructive pulmonary disease, gastro-oesophageal reflux disease, iron-deficiency anaemia |
| Patient 11 | 83 | F | Total hip replacement | 01/04/2019 | Failed fracture fixation | hypothyroid, hypertension, osteoarthritis |
| Patient 12 | 84 | F | Total hip replacement | 12/03/2019 | Osteoarthritis | ischaemic heart disease, type II diabetes mellitus, asthma, hypertension, peripheral neuropathy, venothromboembolism, vitamin D deficiency, B12 and folate deficiency |
| Patient 13 | 88 | F | Total hip replacement | 13/03/2019 | Osteoarthritis | type II diabetes mellitus, chronic kidney disease, atrial fibrillation, diverticular disease, hypertension |

***Supplementary Table 2.***

*Table showing the number of MSCs isolated, the number where mtDNA was successfully amplified and included in mtDNA NGS and the number of cells per sample passing bioinformatic QC. In total 99 cells, across 13 patients were taken forward for analysis, ensuring at least >3 cells were available per patient.*

| **Sample** | **Seq Code** | **MSCs**  **Isolated** | **MSCs**  **Successfully PCR’d** | **MSCs Included in NGS** | **MSCs Passing NGS QC** |
| --- | --- | --- | --- | --- | --- |
| Patient 1 | PB | 10 | 10 (100%) | 10 | 7 (70%) |
| Patient 2 | 25 | 11 | 11 (100%) | 11 | 8 (73%) |
| Patient 3 | 6 | 10 | 10 (100%) | 10 | 3 (30%) |
| Patient 4 | 1 | 10 | 10 (100%) | 10 | 9 (90%) |
| Patient 5 | 61 | 10 | 10 (100%) | 10 | 9 (90%) |
| Patient 6 | 10 | 11 | 11 (100%) | 11 | 7 (64%) |
| Patient 7 | 9 | 12 | 12 (100%) | 12 | 8 (66%) |
| Patient 8 | 8 | 12 | 12 (100%) | 12 | 4 (33%) |
| Patient 9 | 78 | 20 | 20 (100%) | 20 | 11 (55%) |
| Patient 10 | 7 | 10 | 10 (100%) | 10 | 8 (80%) |
| Patient 11 | 4 | 10 | 10 (100%) | 10 | 7 (70%) |
| Patient 12 | 2 | 10 | 10 (100%) | 10 | 8 (73%) |
| Patient 13 | 3 | 10 | 10 (100%) | 10 | 10 (100%) |
|  | ***Total*** | ***146*** | ***146*** | ***146*** | ***99*** |

***Supplementary Table 3***

*Table showing the percentage of the mtDNA genome ( aligned to rCRS or GenBank ID NC_012920.1) covered by at least 1500x in each cell in each patient. Overall, 47 cells were removed due to low coverage (highlighted in red).*

|  |  |  | **mtDNA (rCRS or GenBank ID NC_012920.1) percentage bases covered at depth >1500x** | | | | | | | | | | | | | | | | | | | |
| --- | --- | --- | --- | --- | --- | --- | --- | --- | --- | --- | --- | --- | --- | --- | --- | --- | --- | --- | --- | --- | --- | --- |
| **Sample** | **Seq Code** | **Cells**  **Passing QC** | **1** | **2** | **3** | **4** | **5** | **6** | **7** | **8** | **9** | **10** | **11** | **12** | **13** | **14** | **15** | **16** | **17** | **18** | **19** | **20** |
| Patient 1 | PD | 7 (70%) | 100 | 100 | 85.2 | 100 | 100 | 100 | 100 | 79.2 | 100 | 63.4 |  |  |  |  |  |  |  |  |  |  |
| Patient 2 | 25 | 8 (73%) | 100 | 100 | 100 | 60.5 | 78.2 | 80.5 | 100 | 100 | 100 | 100 | 100 |  |  |  |  |  |  |  |  |  |
| Patient 3 | 6 | 3 (30%) | 79.2 | 75.6 | 78.3 | 100 | 100 | 100 | 65.8 | 62.5 | 80.5 | 88.6 |  |  |  |  |  |  |  |  |  |  |
| Patient 4 | 1 | 9 (90%) | 100 | 100 | 100 | 100 | 100 | 100 | 100 | 100 | 90.2 | 100 |  |  |  |  |  |  |  |  |  |  |
| Patient 5 | 61 | 9 (90%) | 100 | 100 | 100 | 80.0 | 100 | 100 | 100 | 100 | 100 | 100 |  |  |  |  |  |  |  |  |  |  |
| Patient 6 | 10 | 7 (70%) | 100 | 82.8 | 90.6 | 100 | 82.8 | 78.2 | 100 | 100 | 100 | 100 | 100 |  |  |  |  |  |  |  |  |  |
| Patient 7 | 9 | 8 (73%) | 78.2 | 64.6 | 80.2 | 100 | 100 | 90.6 | 100 | 100 | 100 | 100 | 100 | 100 |  |  |  |  |  |  |  |  |
| Patient 8 | 8 | 4 (10%) | 75.2 | 62.2 | 58.2 | 60.3 | 75.6 | 100 | 72.6 | 68.0 | 100 | 90.1 | 100 | 100 |  |  |  |  |  |  |  |  |
| Patient 9 | 78 | 11 (55%) | 100 | 100 | 89.8 | 90.4 | 100 | 100 | 100 | 86.2 | 100 | 100 | 65.4 | 100 | 70.2 | 80.4 | 88.6 | 100 | 90.2 | 100 | 100 | 88.4 |
| Patient 10 | 7 | 8 (80%) | 80.4 | 100 | 100 | 100 | 100 | 100 | 100 | 90.2 | 100 | 100 |  |  |  |  |  |  |  |  |  |  |
| Patient 11 | 4 | 7 (70%) | 100 | 100 | 80.2 | 100 | 100 | 100 | 90.2 | 100 | 86.4 | 100 |  |  |  |  |  |  |  |  |  |  |
| Patient 12 | 2 | 8 (73%) | 100 | 100 | 100 | 100 | 100 | 78.2 | 100 | 100 | 100 | 92.0 |  |  |  |  |  |  |  |  |  |  |
| Patient 13 | 3 | 10 (100%) | 100 | 100 | 100 | 100 | 100 | 100 | 100 | 100 | 100 | 100 |  |  |  |  |  |  |  |  |  |  |

***Supplementary Table 4***

*Table showing the mtDNA haplogroup, number and position of homoplasmic (heteroplasmy fraction, of HF >98%) and heteroplasmic (HF<98% and >2%) variants detected in each consensus sample sequenced from each patient. All samples were of European ancestry (e.g., haplogroups: H, V, J, T, U, K, W, X or I).*

| **Sample** | **Seq Code** | **mtDNA**  **Haplogroup** | **N Consensus**  **Homoplasmies (HF >98%)** | **N Consensus**  **Heteroplasmies (HF <98% and >2%)** |
| --- | --- | --- | --- | --- |
| Patient 1 | PD | H2a1g | 7 (263G, 750G, 951A, 8065A, 8850G, 15326G, 16354T) | 1 ( 10939, 2.9%) |
| Patient 2 | 25 | H2a2a | 7 (1438G, 6776C, 8860G, 8950A, 10754C, 15326G, 16519C) | 4 (11603G, 2.1%; 11606G, 2.3%; 13407G, 2.3% 16129A, 38.2%) |
| Patient 3 | 6 | T2b3b | 32 (263G, 709A, 750G, 930A, 1438G, 1888A, 2706G, 4216C, 4769G, 4917G, 5147A, 7028T, 8697A, 8860G, 10463C, 10750G, 11251G, 11719A, 11812G, 13722G, 14233G, 14766T, 14905A, 15326G, 15452A, 15607G, 15928A, 16126C, 16294T, 16296T, 16304C, 16519C) | 1 (13368A, 94%) |
| Patient 4 | 1 | HV0+195*I | 14 (64T, 195C, 263G, 750G, 1438G, 2280T, 2706G, 3663G, 4769G, 7028T, 8860G, 8862T, 15326G, 16168T) | 0 |
| Patient 5 | 61 | K1a4a1 | 32 (497T, 1189C, 1438G, 1811G, 2706G, 3480G, 4295G, 4769G, 6260A, 7028T, 8860G, 9055A, 9377G, 9698C, 10550G, 11299C, 11467G, 11485C, 11719A, 11840T, 12308G, 12372A, 13740C, 14167T, 14766T, 14798C, 15326G, 15884A, 16224C, 16245T, 16311C, 16519C) | 7 (10908A, 3.3%; 7162A, 3.4%; 13020C, 3.7%; 13062T, 3.9%; 3576T, 4.9%; 3438A, 5.1%; 13056T, 5.8%) |
| Patient 6 | 10 | K2b1a1 | 32 (263G, 750G, 1438G, 1811G, 2217T, 2706G, 3480G, 4769G, 5231A, 7028T, 8860G, 9055A, 9698C, 9716C, 10550G, 11299C, 11467G, 11719A, 11869A, 12308G, 12372A, 13135A, 14037G, 14167T, 14766T, 14798C, 15326G, 16222T, 16224C, 16270T, 16311C, 16519C) | 1 (13612G, 4.6%) |
| Patient 7 | 9 | U5a1b1 | 27 (263G, 1438G ,2706G, 3197C, 3663G, 4769G, 5263T, 7028T, 8860G, 9477A, 9667G, 11467G, 11719A ,12308G, 12358G, 12372A, 13617C, 14766T, 14793G, 15218G, 15326G, 16192T, 16256T, 16270T, 16287T ,16291T, 16399G) | 2 (13635, 7.9%; 14470 8.8%) |
| Patient 8 | 8 | N1a1b (I3) | 25 (73G, 152C, 199C, 204C, 207A, 239C, 250C, 263G, 750G, 1438G, 1719A, 2706G, 4529T, 4769G, 7028T, 8251A, 8860G, 10034C, 10238C, 10398G, 11719A, 12501A, 12705T, 13780G, 14766T, 15043A, 15326G, 15924G, 16086C, 16129A, 16223T, 16287T, 16319A, 16391A, 16519C) | 3 (3576T, 2.4%; 11212T, 2.1%; 11986T, 2.1%) |
| Patient 9 | 78 | H1a3a | 11 (235G, 263G, 750G, 1438G, 3010A, 8860G, 14978G, 15326G, 16051G, 16162G, 16519C) | 3 (3480G, 2.1%; 3557A, 5.2%; 5855T, 11.3%) |
| Patient 10 | 7 | H1a6 | 13 (73G, 151T, 263G, 1438G, 3010A, 4769G, 7073G, 8860G, 11893G, 15326G, 16162G, 16189C, 16519C) | 1 (6720G, 4.1%) |
| Patient 11 | 4 | T2f2 | 31 (263G, 709A, 1438G, 1888A, 2706G, 4216C, 4769G, 4917G, 7028T, 8270T, 8697A, 8854A, 8860G, 10463C, 11251G, 11719A, 11812G, 13368A, 14233G, 14766T, 14905A, 15326G, 15452A, 15607G, 15928A, 16126C, 16189C, 16278T, 16294T, 16296T, 16519C) | 1 (6126G, 24.8%) |
| Patient 12 | 2 | J1b1a1 | 32 (242T, 263G, 295T, 489C, 1438G, 2158C, 2706G, 3010A, 4216C, 4769G, 5460A, 7028T, 8269A, 8557A, 8752G, 8860G, 9221G, 10398G, 11251G, 11719A, 12007A, 12612G, 13708A, 14766T, 15326G, 15452A, 16069T, 16126C, 16145A, 16172C, 16222T, 16261T) | 2 (6723A, 21.6%; 7110C, 14.8%) |
| Patient 13 | 3 | HV0f | 14 (195C, 228A, 263G, 750G, 1438G, 2706G, 4769G, 7028T, 8706G, 8860G, 14323A, 15326G, 15721C, 16298C) | 4 (1411A, 28.4%; 4966A, 11.1%; 7241G, 2.7%; 15572, 3.2%) |

***Supplementary Table 5***

*Table showing the number of somatic heteroplasmic variants (HF>0.02/<0.98) detected in each MSC across each patient sample.*

|  |  |  | **mtDNA (rCRS or GenBank ID NC_012920.1) percentage bases covered at depth >1500x** | | | | | | | | | | | | | | | | | | | |
| --- | --- | --- | --- | --- | --- | --- | --- | --- | --- | --- | --- | --- | --- | --- | --- | --- | --- | --- | --- | --- | --- | --- |
| **Sample** | **Seq Code** | **Cells**  **Passing QC** | **1** | **2** | **3** | **4** | **5** | **6** | **7** | **8** | **9** | **10** | **11** | **12** | **13** | **14** | **15** | **16** | **17** | **18** | **19** | **20** |
| Patient 1 | PD | 7 (70%) | 22 | 20 | - | 29 | 24 | 28 | 21 | - | 24 | - |  |  |  |  |  |  |  |  |  |  |
| Patient 2 | 25 | 8 (73%) | 19 | 16 | 20 | - | - | - | 18 | 33 | 29 | 24 | 21 |  |  |  |  |  |  |  |  |  |
| Patient 3 | 6 | 3 (30%) | - | - | - | 29 | 21 | 12 | - | - | - | - |  |  |  |  |  |  |  |  |  |  |
| Patient 4 | 1 | 9 (90%) | 27 | 34 | 35 | 37 | 31 | 36 | 26 | 31 | - | 27 |  |  |  |  |  |  |  |  |  |  |
| Patient 5 | 61 | 9 (90%) | 20 | 22 | 40 | - | 29 | 13 | 16 | 22 | 34 | 23 |  |  |  |  |  |  |  |  |  |  |
| Patient 6 | 10 | 7 (70%) | 22 | - | - | 28 | - | - | 22 | 11 | 24 | 31 | 22 |  |  |  |  |  |  |  |  |  |
| Patient 7 | 9 | 8 (73%) | - | - | - | 27 | 23 | - | 28 | 16 | 25 | 21 | 25 | 23 |  |  |  |  |  |  |  |  |
| Patient 8 | 8 | 4 (10%) | - | - | - | - | - | 29 | - | - | 13 | - | 23 | 25 |  |  |  |  |  |  |  |  |
| Patient 9 | 78 | 11 (55%) | 18 | 27 | - | - | 29 | 12 | 30 | - | 31 | 26 | - | 24 | - | - | - | 20 | - | 20 | 27 | - |
| Patient 10 | 7 | 8 (80%) | - | 36 | 18 | 36 | 12 | 15 | 26 | - | 24 | 30 |  |  |  |  |  |  |  |  |  |  |
| Patient 11 | 4 | 7 (70%) | 23 | 27 | - | 24 | 25 | 20 | - | 37 | - | 20 |  |  |  |  |  |  |  |  |  |  |
| Patient 12 | 2 | 8 (73%) | 15 | 28 | 27 | 25 | 36 | - | 37 | 21 | 31 | - |  |  |  |  |  |  |  |  |  |  |
| Patient 13 | 3 | 10 (%) | 22 | 22 | 33 | 28 | 26 | 27 | 32 | 24 | 26 | 29 |  |  |  |  |  |  |  |  |  |  |

***Supplementary Table 6***

*Table showing the proportions of substitution types amongst the 2,477 somatic heteroplasmies detected.*

*Stranded bias?*

| ***Substitution*** | ***Count*** | ***%*** |
| --- | --- | --- |
| T > C | 841 | 33.95 |
| A > G | 737 | 29.75 |
| C > T | 512 | 20.67 |
| G > A | 226 | 9.12 |
| A > C | 48 | 1.94 |
| T > G | 37 | 1.49 |
| C > A | 29 | 1.17 |
| T > A | 17 | 0.69 |
| G > T | 10 | 0.4 |
| A > T | 10 | 0.4 |
| C > G | 9 | 0.36 |
| G > C | 1 | 0.04 |
| Total | 2,477 | 100 |
